# A soft X-ray plane-grating monochromator optimized for elliptical dipole radiation from modern sources

**DOI:** 10.1107/S1600577515010826

**Published:** 2015-07-14

**Authors:** Torsten Kachel, Frank Eggenstein, Rolf Follath

**Affiliations:** a Institute for Methods and Instrumentation in Synchrotron Radiation Research, Helmholtz-Zentrum Berlin für Materialien und Energie, Albert-Einstein-Strasse 15, 12489 Berlin, Germany; b Institute for Nanometer Optics and Technology, Helmholtz-Zentrum Berlin für Materialien und Energie, Albert-Einstein-Strasse 15, 12489 Berlin, Germany; c Beamline Optics Group, Paul Scherrer Institute, 5232 Villigen, Switzerland

**Keywords:** soft X-rays, plane-grating monochromator, PGM, elliptical polarization, dipole radiation

## Abstract

A new but yet well proven way of making elliptically polarized dipole radiation from the BESSY II storage ring applicable to the SX700-type collimated plane-grating monochromator PM3 is described. It is shown that due to the limited vertical acceptance of the grating a simple use of vertical apertures is not possible in this case. Rather, deflecting the beam upwards or downwards by rotating the vertically collimating toroidal mirror M1 around the light axis leads to excellent performance. The resulting detuning of the photon energy can be taken into account by a readjustment of the monochromator internal plane mirror M2. The energy resolution of the beamline is not affected by the non-zero ‘roll’ of the collimating mirror.

## Abstract 2

The utilization of elliptical dipole radiation in a collimated plane-grating monochromator at BESSY II is described.

## 1. Introduction   

For more than two decades the use of circularly polarized soft X-rays has developed as a major tool for studying magnetic thin films and surfaces. Namely, the application of X-ray magnetic circular dichroism (XMCD) (Schütz *et al.*, 1987 ▸; Chen *et al.*, 1990 ▸; Baumgarten *et al.*, 1990 ▸; for an extensive overview see, for example, Stöhr & Siegmann, 2006 ▸) offers a wide field of experimental applications. Whenever the orientation between the *k*-vector of the impinging elliptically polarized soft X-rays and the sample magnetization vector is changed, a variation of the absorption coefficient is observed. Today this fact is well known and understood (Thole *et al.*, 1985 ▸). The XMCD effect can be seen in numerous experimental applications such as, for example, photoemission spectroscopy, total electron yield spectroscopy, X-ray absorption, transmission and reflectivity measurements.

Therefore, the need for beamlines delivering elliptically polarized synchrotron radiation (SR) and, as a consequence, the number of such beamlines has increased significantly. An important improvement was the extension of the photon energy range from low-energy (*h*ν ≤ 25 eV) normal-incidence monochromators (Schäfers *et al.*, 1986 ▸) to grazing-incidence plane-grating (PGM) or spherical-grating monochromators and the implication of crossed (Bahrdt *et al.*, 1992 ▸) or elliptical (Sasaki *et al.*, 1992 ▸) undulators delivering about 100 times more photon flux than dipole sources.

Along with these improvements, considerable progress has been made in soft X-ray beamline design. This holds pre­dominantly concerning properties like brilliance, energy tunability, energy resolution and focal spot size. In particular, and important in the context of this paper, the development of the so-called ‘collimated PGM’ (cPGM) (Follath & Senf, 1997 ▸) has been established a significant design improvement. A photon energy resolution down to the diffraction limit and tunability of the *c*_ff_-value that allows for easy energy calibration and higher-order suppression are only a few of its features.

It is understood that the combination of both using elliptically polarized dipole radiation and applying a cPGM is not for free unless additional effort is made. However, here we will outline that a simple rotation of the collimation mirror can combine both objectives.

## 2. PM3 beamline layout   

**Figure 1:**
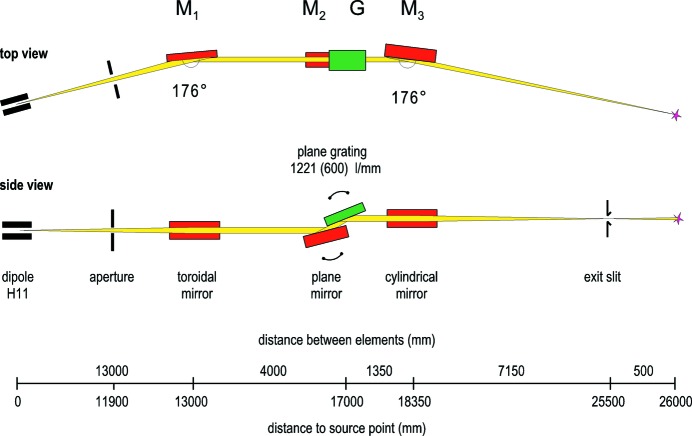
Beamline layout of PM3 at BESSY II. The dipole source is on the left, the exit slit on the right. The toroidal mirror M1 serves for vertical (sagittal) collimation of the synchrotron light.

The beamline layout follows the collimated design that was implemented in several beamlines at BESSY II previously (Follath, 2001 ▸). Fig. 1 ▸ shows the design of PM3. It consists of four optical elements.

The toroidal mirror M1, at 13000 mm from the source, collimates the divergent beam vertically (sagittally). Horizontally (meridionally) it focuses the beam with unit magnification at 26000 mm. M2 and the grating are plane elements in the standard Zeiss SX700 configuration. This is the same, partly upgraded, monochromator that has been in operation at BESSY I as ‘SX700-III’ (Petersen *et al.*, 1993 ▸). The grating is located at 17000 mm from the source. All experimental data and calculations in this paper are related to the high-resolution 1221 l mm^−1^ grating.

Because of its restricted length of 120 mm and typical incidence angles α of less than 5° the grating has the smallest vertical acceptance of all four optical elements and defines therefore the vertical acceptance of the beamline.

**Figure 2:**
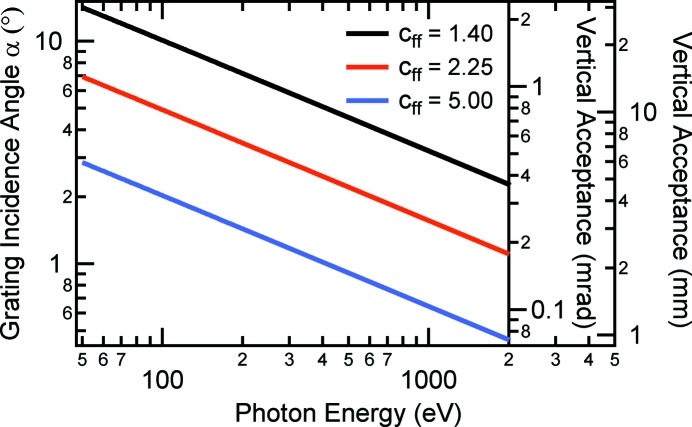
Vertical acceptance of the BESSY II PM3 grating for varying *c*_ff_ values.

In Fig. 2 ▸ we present calculated values for the incidence angle α as well as the vertical acceptance in mm and in mrad of the 120 mm-long grating. The values are parameterized for typical *c*_ff_ values: *c*_ff_ = 2.25 (standard operation), *c*_ff_ = 1.4 (higher-order suppression mode) and *c*_ff_ = 5 (high-resolution mode). We note that the acceptances in mm and mrad are connected through the 13000 mm entrance arm length of M1.

## 3. Source characteristics of BESSY II dipoles   

It is well known that SR from dipole magnets is polarized (Schwinger, 1949 ▸). For a further discussion we define the off-plane viewing angle ψ and the angle of acceptance Δψ. Both quantities might be defined by vertical apertures. The position of the center of acceptance defines ψ whereas the size is a measure for Δψ. At ψ = 0, *i.e.* within the storage ring plane, the polarization is linear with the polarization vector lying in the horizontal plane. At finite ψ the polarization is right- or left-handed elliptical for negative and positive viewing angles, respectively.

**Figure 3:**
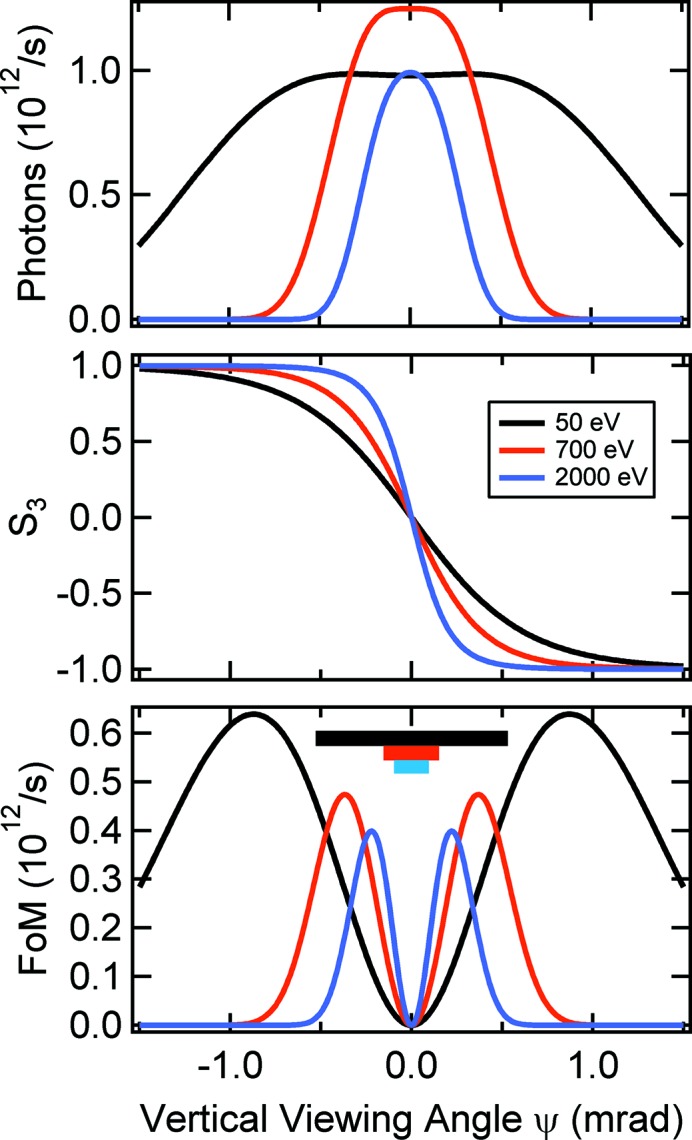
Calculated flux (top panel), ellipticity (center) and figure of merit (FoM, bottom) for *h*ν = 50, 700 and 2000 eV from BESSY II dipoles. The bars in the bottom panel indicate the vertical acceptance of the PM3 grating at the color-coded photon energies in standard operation (*c*_ff_ = 2.25).

For given storage ring parameters, *i.e.* fixed electron energy and magnetic dipole field, the vertical width of the emission cone is a function of the photon energy only. For the BESSY II case and selected photon energies the situation appears as shown in Fig. 3 ▸. The curves have been calculated with the *REFLEC* software (Schäfers & Krumrey, 1996 ▸). All panels show vertical distributions for a set of different photon energies which are near the lowest and highest accessible photon energies of the PM3 and an intermediate one of interest (Fe 2*p* absorption edge).

In the calculation we applied vertical acceptance angles Δψ that are determined by the vertical acceptance of the grating in standard operation (*c*_ff_ = 2.25). The horizontal beamline acceptance is determined by the 980 mm optical length of M1 which refers to 3.42 mrad constantly. In the top panel of Fig. 3 ▸ we present the photon flux *I*. We notice that the FWHMs of the vertical emission cones range from about 0.6 to 2.5 mrad. The circular polarization *S*_3_ (which should more precisely be called the Stokes–Poincaré parameter) in the center panel of Fig. 3 ▸ behaves likewise. However, rather than flux *I* and circular polarization *S*_3_, the quantity of interest for optimal experimental conditions is the figure of merit (FoM) (Petersen *et al.*, 1993 ▸), defined as
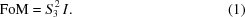
The FoM is depicted in the bottom panel of Fig. 3 ▸. We observe maxima at ψ ≃ 0.2 (2000 eV), 0.35 (700 eV) and 0.85 mrad (50 eV). As stated above, these values are too large to be accepted by the grating when using the cPGM standard alignment (see Fig. 2 ▸). This is illustrated by the colored bars indicating the photon-energy-dependent acceptance of the grating. In contrast, the collimation mirror M1 with a vertical optical active width of 40 mm (= 3.1 mrad) covers all necessary viewing angles ψ. From the S-shaped ellipticity curves in the center panel of Fig. 3 ▸ it is evident that, in the case of larger viewing angles, errors in ψ have little effect on *S*_3_. In contrast, for π-light the setting of ψ is much more critical. In other words, it is hardly possible to achieve π-polarization precisely.

By comparing Figs. 2 ▸ and 3 ▸ it becomes obvious that for *c*_ff_ = 2.25 we find an incidental very good matching between the required (Fig. 2 ▸) and naturally given (Fig. 3 ▸) beamline acceptance ranging from 0.2 to 1 mrad. Therefore, the application of vertical entrance apertures is not required. For higher *c*_ff_ values the acceptance becomes smaller and, thus, it is always possible to select radiation of high ellipticity. Only in the higher-order suppression mode *c*_ff_ = 1.4, where the acceptance ranges from 0.4 to 2 mrad, are additional apertures needed to select elliptically polarized SR with high FoM. Obviously, the same statement holds true when operating the monochromator in outside (negative) diffraction order, where the acceptance is even larger than for *c*_ff_ = 1.4.

## 4. Changing the viewing angle ψ by rotation of the collimation mirror   

Up to now we have mainly discussed the acceptance Δψ. However, the main issue of the setup is the viewing angle ψ. Fig. 3 ▸ shows that SR of high FoM cannot be accepted by the grating when using the standard alignment. In the past years different principles have been applied to steer the desired part of the SR cone onto the grating, namely employing additional vertically deflecting mirrors (Petersen *et al.*, 1993 ▸) or implementing a vertical ‘bump’ to the electron beam within the dipole, *i.e.* steering the ‘storage ring plane’ (Hunter Dunn *et al.*, 2004 ▸; Raabe *et al.*, 2008 ▸). We note that the latter principle of steering the electron beam has been tested but found to be not applicable at the third-generation storage ring BESSY II (Kachel & Feikes, 2000 ▸). Instead, in PM3 we used the roll of M1, *i.e.* the rotation of M1 around the axis of the incoming light, to steer the required part of the synchrotron beam into the beamline. This is an elegant way to avoid additional mirrors. We refer to this degree of freedom as *R*_*z*_, denoting the rotation around *z* (= light axis) of the collimation mirror. Changing *R*_*z*_ leads to an upward or downward reflection of the SR. It can be tuned such that the maximum FoM lies in the grating center. For all-day operation we use seven predefined rotation angles: one for linearly polarized and three each for left- and right-handed elliptical polarization. Table 1 ▸ gives an overview of the related parameters. The fact that the nominal *R*_*z*_ values are not fully symmetric is caused by cross-talking of the actuators for the different degrees of freedom. The asymmetry in the deflection angles arises because the grating center is not perfectly hit under all conditions. It is seen that in our case a free *R*_*z*_-rotation of ±2° is sufficient. We note that for finite *R*_*z*_ a slight correction of the horizontal deflection is also required.

A side effect of the roll variation of M1 is the change of the incident angle on M2 and the grating that leads to a detuning of the photon energies. This is compensated by a readjustment of the plane mirror M2 rotation. As a result, the photon energy at the sample position stays unaltered after a polarization change. As the amount of M2-rotation depends only on the roll-angle of M1 it is independent of the photon energy. The offset angles for M2 have been calculated and experimentally determined for each polarization setting using N_2_ gas absorption spectra.

**Figure 4:**
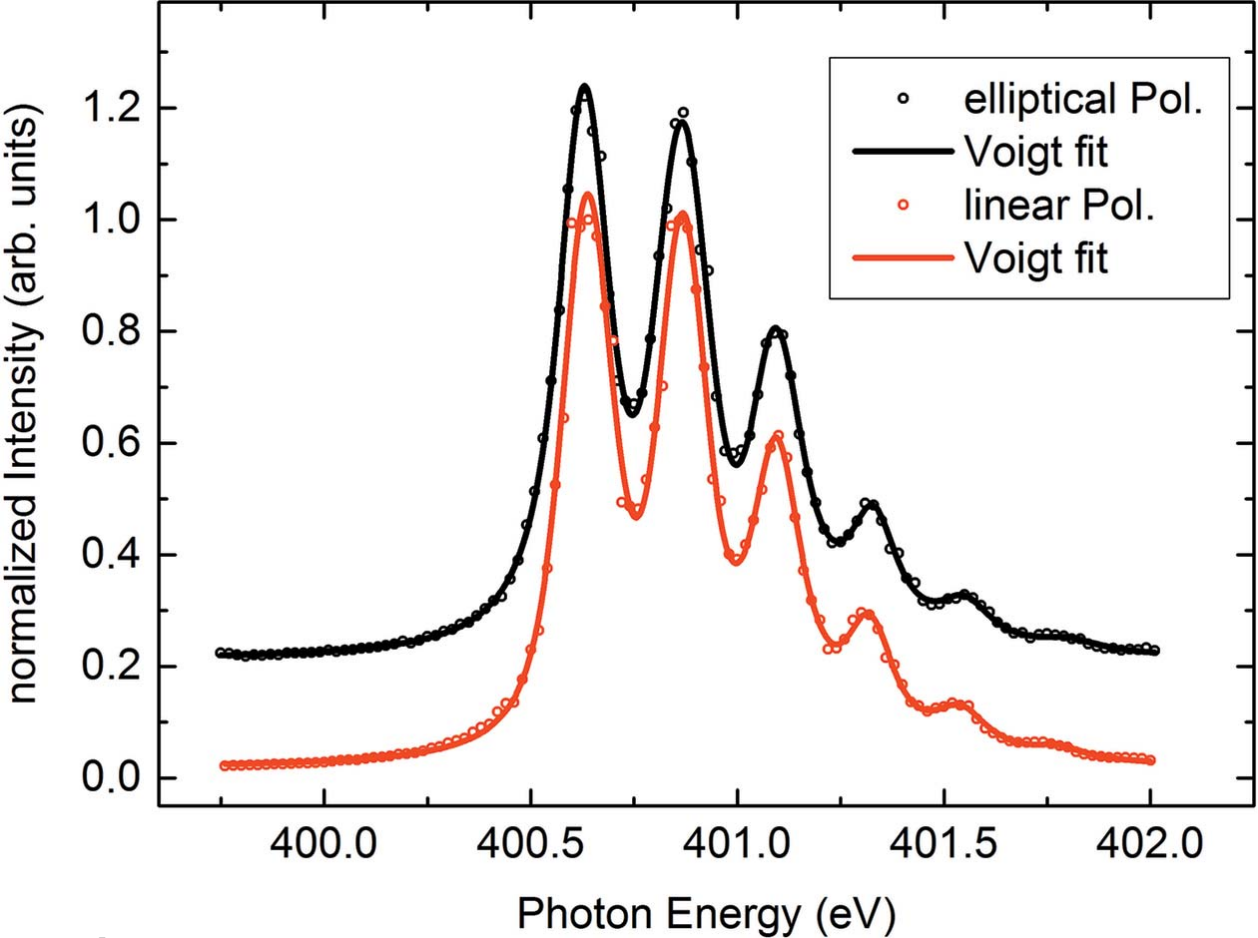
Ion yield at the N 1*s* absorption line of N_2_. Black: σ^+^-polarization (with vertical offset for better visibility); red: π-polarization. Circles: experiment; lines: least-squares fits of six Voigt peak profiles.

The energy resolution of the beamline has been measured by gas absorption spectroscopy. The ion yield of the He 2,−1_4_ peak in the Rydberg series gives a total line width of 1.9 meV at about 64 eV photon energy. This corresponds to an energy resolution
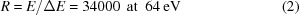
in an optimal case using *c*_ff_ = 5. Concerning our new principle of polarization tuning it might rather be relevant to show how the change of *R*_*z*_ affects the resolving power of the beamline. This is due to the fact that a detuning of the M1 roll might well be interpreted as an on-purpose ‘misalignment’ of a critical optical beamline element. The experiment, however, gave unambiguous proof of a stable high performance under the conditions listed in Table 1 ▸. In Fig. 4 ▸ we present N 1*s* ion yield spectra of the N_2_ absorption obtained with linearly and elliptically polarized SR from PM3. We conclude that no obvious degradation of the energy resolution is observed in the case of finite roll *R*_*z*_ for elliptically polarized SR.

Under typical operation conditions the beamline delivers an experimentally determined ellipticity of 92% at the Fe *L*_3_ edge (*h*ν = 707 eV). The values are similar for the other transition metal 2*p* absorption edges. In contrast, photon energies below about 100 eV are rarely used. This is due to the fact that in this energy range only few absorption edges of interest in XMCD work exist and that the degree of ellipticity might be easily obscured by depolarization effects of the beamline (Bahrdt *et al.*, 2010 ▸). But it has been shown that, even at the Pt 4*f* absorption edge at about 72 eV, PM3 delivers a high degree of circular polarization and precision of XMCD asymmetries much better than 0.1% (Honolka *et al.*, 2009 ▸). The highest photon energy used so far with elliptical polarization was 1853 eV for resonant excitation of the Si 1*s* edge. In that experiment, induced magnetic moments in Heusler-like Fe_3_Si were studied (Antoniak *et al.*, 2012 ▸). Further prominent publications from the PM3 beamline can be found in additional references (Valencia *et al.*, 2011 ▸; Radu *et al.*, 2012 ▸; Antoniak *et al.*, 2011 ▸; Mishra *et al.*, 2009 ▸; Sanyal *et al.*, 2010 ▸).

## 5. Summary   

We have shown that a state-of-the-art high-resolution collimated plane-grating monochromator can exploit elliptically polarized dipole radiation from a third-generation storage ring without additional mirrors or electron beam steering. A simple rotation *R*_*z*_ of the collimation mirror (M1) around the axis of the incoming SR beam is sufficient to direct the elliptically polarized part of the dipole emission cone onto the grating. The resulting energy shift can be compensated by a *R*_*z*_-dependent offset in the plane mirror (M2) rotation angle. No degradation of the energy resolution caused by the steering with M1 could be observed.

## Figures and Tables

**Table 1:** M1 rotation around *R*_*z*_ in seven steps The column ‘M2 offset angle (°)’ denotes half the vertical beam deflection behind M1.

Naming	Polarization	*R*_*z*_ (µrad)	M2 offset angle (°)	Energy range (eV)
+1.2	σ^+^	32279	0.06150	<100
+0.8	σ^+^	21862	0.04100	100–1000
+0.4	σ^+^	11445	0.02060	>1000
Pi	π	0	0.00000	Full range
−0.4	σ^−^	−9388	−0.02050	>1000
−0.8	σ^−^	−19805	−0.04114	100–1000
−1.2	σ^−^	−30222	−0.06190	<100

## References

[bb1] Antoniak, C., Gruner, M. E., Spasova, M., Trunova, A. V., Römer, F. M., Warland, A., Krumme, B., Fauth, K., Sun, S., Entel, P., Farle, M. & Wende, H. (2011). *Nat. Commun.* **2**, 528. 10.1038/ncomms1538 22068595

[bb2] Antoniak, C., Herper, H. C., Zhang, Y. N., Warland, A., Kachel, T., Stromberg, F., Krumme, B., Weis, C., Fauth, K., Keune, W., Entel, P., Wu, R. Q., Lindner, J. & Wende, H. (2012). *Phys. Rev. B*, **85**, 214432.

[bb3] Bahrdt, J., Follath, R., Frentrup, W., Gaupp, A., Scheer, M., Garrett, R., Gentle, I., Nugent, K. & Wilkins, S. (2010). *AIP Conf. Proc.* **1234**, 335–338.

[bb4] Bahrdt, J., Gaupp, A., Gudat, W., Mast, M., Molter, K., Peatman, W. B., Scheer, M., Schroeter, Th. & Wang, Ch. (1992). *Rev. Sci. Instrum.* **63**, 339–342.

[bb5] Baumgarten, L., Schneider, C. M., Petersen, H., Schäfers, F. & Kirschner, J. (1990). *Phys. Rev. Lett.* **65**, 492–495. 10.1103/PhysRevLett.65.492 10042934

[bb23] Chen, C. T., Sette, F., Ma, Y. & Modesti, S. (1990). *Phys. Rev. B*, **42**, 7262(R). 10.1103/physrevb.42.7262 9994861

[bb6] Follath, R. (2001). *Nucl. Instrum. Methods Phys. Res. A*, **467**–**468**, 418–425.

[bb7] Follath, R. & Senf, F. (1997). *Nucl. Instrum. Methods Phys. Res. A*, **390**, 388–394.

[bb8] Honolka, J., Lee, T. Y., Kuhnke, K., Enders, A., Skomski, R., Bornemann, S., Mankovsky, S., Minár, J., Staunton, J., Ebert, H., Hessler, M., Fauth, K., Schütz, G., Buchsbaum, A., Schmid, M., Varga, P. & Kern, K. (2009). *Phys. Rev. Lett.* **102**, 067207. 10.1103/PhysRevLett.102.067207 19257632

[bb9] Hunter Dunn, J., Hahlin, A., Karis, O., Arvanitis, D., LeBlanc, G., Andersson, Å. & Lindgren, L.-J. (2004). *AIP Conf. Proc.* **705**, 65.

[bb10] Kachel, T. & Feikes, J. (2000). Unpublished results.

[bb11] Mishra, S. K., Radu, F., Dürr, H. A. & Eberhardt, W. (2009). *Phys. Rev. Lett.* **102**, 177208. 10.1103/PhysRevLett.102.177208 19518827

[bb12] Petersen, H., Willmann, M., Schäfers, F. & Gudat, W. (1993). *Nucl. Instrum. Methods Phys. Res. A*, **333**, 594–598.

[bb13] Raabe, J., Tzvetkov, G., Flechsig, U., Böge, M., Jaggi, A., Sarafimov, B., Vernooij, M. G. C., Huthwelker, T., Ade, H., Kilcoyne, D., Tyliszczak, T., Fink, R. H. & Quitmann, C. (2008). *Rev. Sci. Instrum.* **79**, 113704. 10.1063/1.3021472 19045892

[bb14] Radu, F., Abrudan, R., Radu, I., Schmitz, D. & Zabel, H. (2012). *Nat. Commun.* **3**, 715. 10.1038/ncomms1728 22395606

[bb15] Sanyal, B., Antoniak, C., Burkert, T., Krumme, B., Warland, A., Stromberg, F., Praetorius, C., Fauth, K., Wende, H. & Eriksson, O. (2010). *Phys. Rev. Lett.* **104**, 156402. 10.1103/PhysRevLett.104.156402 20482001

[bb16] Sasaki, S., Miyata, K. & Takada, T. (1992). *Jpn. J. Appl. Phys.* **31**, L1794–L1796.

[bb17] Schäfers, F. & Krumrey, M. (1996). *Technischer Ber.* pp. 201 Berliner Elektronenspeicherring-Gesellschaft für Synchrotronstrahlung.

[bb18] Schäfers, F., Peatman, W., Eyers, A., Heckenkamp, Ch., Schönhense, G. & Heinzmann, U. (1986). *Rev. Sci. Instrum.* **57**, 1032.

[bb19] Schwinger, J. (1949). *Phys. Rev.* **75**, 1912–1925.

[bb22] Schütz, G., Wagner, W., Wilhelm, W., Kienle, P., Zeller, R., Frahm, R. & Materlik, G. (1987). *Phys. Rev. Lett.* **58**, 737–740. 10.1103/PhysRevLett.58.737 10035022

[bb20] Stöhr, J. & Siegmann, H. C. (2006). *Magnetism: From Fundamentals to Nanoscale Dynamics.* Berlin: Springer.

[bb24] Thole, B. T., van der Laan, G. & Sawatzky, G. A. (1985). *Phys. Rev. Lett.* **55**, 2086–2088. 10.1103/PhysRevLett.55.2086 10032006

[bb21] Valencia, S., Crassous, A., Bocher, L., Garcia, V., Moya, X., Cherifi, R. O., Deranlot, C., Bouzehouane, K., Fusil, S., Zobelli, A., Gloter, A., Mathur, N. D., Gaupp, A., Abrudan, R., Radu, F., Barthélémy, A. & Bibes, M. (2011). *Nat. Mater.* **10**, 753–758. 10.1038/nmat3098 21857674

